# Supplementary material for: Improving on-field decision making using video-based training – A pilot study with young volleyball players
Source: PLoS One. 2025 Dec 9;20(12):e0338523. doi: 10.1371/journal.pone.0338523 (PMC12688115; doi:10.1371/journal.pone.0338523)
Supplement: Attachment — Submitted filename: Response to Reviewers.docx [file pone.0338523.s003.docx]

**Reviewer 1**

The introduction is well-directed and successfully presents the research problem, supported by relevant studies. However, to further strengthen this section, I recommend including references that specifically address the terminology "fast ball sports" and the necessity to process and monitor multiple sources of information from opponents, teammates, and the ball. Adding such references would substantiate the use of this nomenclature.

We appreciate the reviewer’s positive comments about the introduction and advice for improvement. On line 35, We have included a textbook reference (Davids et al., 2002) to support the statement that it is necessary to process and monitor multiple sources of information from opponents, teammates and the ball.

As for our reference to “fast ball sports”, we now realize that this was not the best term to describe sporting situations where it is necessary to monitor teammates. We have therefore changed this term to “team sports” (line 33).

Additionally, in lines 45-47, where it is stated that on-field training for decision making is not simple, not individualized, and often overlooked in favor of technical skills training, it would be beneficial to include references that have discussed or demonstrated a similar focus on technical skills at the expense of decision-making training.

With hindsight, we realize that our comment was somewhat motivated by personal experience. We have revised this sentence (lines 45-46) to reflect the fact there are difficulties with on-field training for decision making, which has led researchers to consider off-field training using video footage.

Methods:

In the methods section, particularly in lines 138-139, where it is mentioned that the number of opponents, court size, number of zones, and number of clips per condition are presented in Table 1, I suggest adding a brief explanation of how the court sizes were determined. This would clarify the rationale behind the experimental setup.

We have clarified in the revised manuscript (lines 133-134) that the volleyball specific aspects of the methods were developed in collaboration with certified coaches to reflect typical volleyball situations.

Furthermore, in lines 166-167, where it is mentioned that the aim was to ensure high-quality passes that would provide all spiking options, it would be important to briefly define what was considered a "high-quality pass" to enhance the understanding of the experimental conditions.

In volleyball, a high-quality pass is a pass that provides adequate time and space and optimal scoring conditions to the spiker. This has been clarified in the text (lines 169-171).

In lines 211-213, where different topics related to difficulty levels are discussed—such as the position of the block relative to the sidelines and the defenders relative to the block—it would be helpful to specify more clearly what criteria were used to assess these positions and what was considered a "bad pass."

We have revised the text (lines 214-221) to clarify by way of examples the different topics discussed. We have replaced reference to a bad pass with reference to a pass that is difficult for the spiker to reach, or does not provide optimal scoring conditions.

Similarly, in lines 214-216, where the intervention aims to improve decision making in both standard and non-standard situations (e.g., bad pass or incorrectly formed block), a clearer definition of what constitutes a "correctly formed block" would strengthen the methodology description.

This has been covered in the revision mentioned above.

Finally, in lines 233-235, where a panel of three expert coaches selected the most optimal zone(s) for decision making, I recommend clarifying the criteria used to determine the "most optimal zone(s)."

We have clarified that the “most optimal zone” is the zone with the highest scoring probability, based on the position of the block and the defenders. This has been clarified in the text (lines 242-244).

Discussion:

Regarding the discussion section, I suggest restructuring it to first state that this was a pilot study, helping readers to appropriately contextualize the findings.

Revised as suggested (line 309).

Furthermore, I recommend organizing the discussion based on the hypotheses presented at the end of the introduction, using them as a framework to guide the interpretation of results.

We have included reference to the hypothesis when presenting the main findings at the start of the discussion (lines 310-313).

Additionally, it would be beneficial to clearly separate the suggestions for future research and the limitations of the current study into distinct final sections, concluding with a concise summary of the key findings and contributions.

We have revised the discussion as suggested (lines 363-378).

**Reviewer 2**

The initial sample size was small (N=20), and only 16 people completed the study in the end. This may affect the generalizability of the results. The article mentions that the original plan was to have N=50, but due to difficulties in actual recruitment, only N=20 was achieved. It is necessary to explain whether a post hoc power analysis was conducted to assess whether the current sample size is sufficient to support the conclusion.

The reviewer raises a relevant point here. The sample size is lower than what the a priori sample size calculation suggested, and this has an effect on the power of our analysis. In line with Lakens (2022), we have performed a sensitivity power analysis to determine the minimum detectable effect size based on the given sample size, alpha level of 0.05 and statistical power of 80%. This analysis reveals that the study has 80% power to detect an effect size of *𝜂_p_² = 0.175* (see plot below), which is greater than the effect size of *𝜂_p_² = 0.04 for* motor execution. Interestingly, the effect size for motor execution is also lower than the critical threshold of 0.25 (partial eta squared = 0.059) that we specified in the a-priori sample size estimation, which indicated a total sample size of 50. As such, we believe it is justified to conclude that there was no significant improvement in this dependent measure by the experimental group.

That said, we agree that small sample size impacts upon the generalisability of the results, which has been acknowledged in a section on the study limitations. Also, when discussing the findings (lines 324-336), we have included a comment on the number of participants showing no change or a decrease in performance. We feel that this provides the reader with a better understanding of the between-participant variability in response to the intervention.

Lakens, D. (2022). Sample Size Justification. Collabra: Psychology,8(1).

https://doi.org/10.1525/collabra.33267

Was the training content of the control group completely unrelated to decision-making ability? It is necessary to provide additional explanations regarding the design logic of the tasks in the control group to avoid potential confounding factors that may indirectly affect decision-making ability.

This was indeed a concern that we considered when developing the video clips for the control group. As such, we designed the tasks and instructions in order to minimize any indirect effect on offensive decision making ability. As outlined in the text, these tasks involved evaluating the quality of the first ball reception, the setter's pass, and the direction of the set-up. The key point, then, is that these tasks required participants to focus on the ball’s flight path rather than on the positioning of blockers and defenders, which is considered critical for offensive decision making. We have clarified this further in the revised text (lines 230-235).

The article mentions "random grouping by teams", but does not specify whether there are any baseline differences among the teams.

On lines 105-106 we have clarified that: “To limit baseline difference between groups, the selected volleyball teams were all playing at the same competition level.” We did refer to lack of baseline differences between the groups for video-based decision making in the original manuscript, but not for the on-field task. We have added this information in the results section of the revised manuscript (lines 289-290 and 300-301).

Although the on-site tests simulate the competition scenarios, they rely on a fixed defensive mode, which may differ from the dynamic complexity of real competitions.

We appreciate the reviewer’s comment and have considered this in a new section on the study limitations (lines 363-378).

Only the retention effect after 4 weeks was evaluated, and there was a lack of long-term follow-up.

Again, we agree and have commented on this in the limitations.

The condition checks for the prerequisite of variance analysis were not mentioned.
Should multiple comparisons be corrected? The article mentioned using Bonferroni correction, but it is necessary to clarify which analyses it is specifically applied to.

Assumptions of normality and homogeneity of variances were checked using QQ plots and Levene’s test. We have not presented these data in the manuscript but we can confirm that assumptions were met, except for the motor execution retention data which indicated unequal variance (p=0.034). Given the robustness of ANOVA and equal sample sizes, combined with the lack of significant interaction effects, we chose not to transform the data or run an alternative test.

We reported in the original manuscript that estimated marginal means were compared with Bonferroni correction. We have clarified the approach in the revised manuscript (lines 276-278), which now reads “Significant interaction effects were further analysed using pairwise comparisons of estimated marginal means with Bonferroni correction.” We would like to note, however, that in the process of confirming our previous posthoc analysis, we have become aware that SPSS does not correctly apply Bonferroni correction for 2x2 interaction effects. This seems to be a known problem, although not widely reported or corrected by SPSS. We have subsequently calculated Bonferroni correction manually, which has changed our findings slightly. The previously reported significant difference from baseline to retention for on-field verbal decision making now has a corrected alpha of p = 0.085). This has been address in the revised manuscript on lines 324-336.
